# Healthcare contact days in cancer survivors relative to those with no cancer history

**DOI:** 10.1007/s00520-026-10884-8

**Published:** 2026-06-16

**Authors:** Helen M. Parsons, Christina C. Newton, Arjun Gupta, W. Dana Flanders, Alpa V. Patel, Clara Bodelon, Charlie Zhong, J. Lee Westmaas, Erika Rees-Punia

**Affiliations:** 1 Division of Health Policy and Management, School of Public Health, University of Minnesota, Minneapolis, MN USA; 2 Department of Population Science, American Cancer Society, Atlanta, GA USA; 3 Department of Hematology, Oncology and Transplantation, School of Medicine, University of Minnesota, Minneapolis, MN USA; 4 Department of Epidemiology, Rollins School of Public Health, Emory University, Atlanta, GA USA; 5 Department of Surveillance & Health Equity Science, American Cancer Society, Atlanta, GA USA

**Keywords:** Healthcare contact, Cancer survivor, Time burden

## Abstract

**Introduction:**

Cancer survivors have unique healthcare needs that require frequent contact with the healthcare system to receive cancer-directed treatment and survivorship care. While there is growing evidence of survivors’ time burden for cancer care, we have less information about the trajectory of this burden across a broad range of cancers relative to those without cancer.

**Methods:**

Using the Cancer Prevention Study-II Nutrition Cohort linked to Medicare claims from 1999–2017, we describe trajectories of and factors associated with healthcare contact after a cancer diagnosis relative to those without cancer. We use logistic regression to examine the relationship between sociodemographic and cancer characteristics and being in the highest quartile of healthcare contact days.

**Results:**

In the month of diagnosis the median percentage of days with healthcare contact spiked at 20% compared with 4% among controls and remained higher than controls throughout the year after diagnosis. In the last year of life, cancer cases had a higher median percentage of contact days vs. controls for all months except for the month of death. The highest levels of healthcare contact were among individuals with lung and hematologic cancers, those receiving chemotherapy and/or radiation, older individuals, females, Non-Hispanic White individuals, those with more comorbidities or people living in metropolitan areas.

**Conclusion:**

We identify substantial healthcare contact among cancer survivors relative to controls after diagnosis. Our findings can serve as a framework for discussion between providers and patients to inform interventions focused on non-value added healthcare contact across the cancer continuum.

**Supplementary Information:**

The online version contains supplementary material available at 10.1007/s00520-026-10884-8.

## Introduction

Over the next two decades, the number of cancer survivors is projected to increase to more than 26 million individuals in the United States [1, 2]. Cancer survivors have unique healthcare needs that require frequent contact with the healthcare system to receive cancer-directed treatment, monitor for cancer recurrence, manage cancer-related toxicities and support health promotion and psychosocial well-being [3, 4]. However, frequent healthcare visits can be time and resource intensive, leading to substantial burden for survivors and their families and take time away from work, family and social pursuits [5–9]. These time burdens, defined broadly as the time resources required to complete necessary cancer-related treatment and tasks that take away from other life responsibilities (e.g., driving to and attending medical appointments), do not exist in isolation [8]. Rather, these time burdens may intersect with and be compounded by the related administrative (e.g., making appointments, completing prior authorization forms) and financial burdens (e.g., medical debt) that accompany cancer treatment and supportive care [8, 10, 11]. The concept of time burden in cancer care is increasingly recognized as an important dimension of treatment burden [12, 13] that, given the growing complexity and extended treatment trajectories of innovative cancer therapies [14], should be examined across the cancer continuum to inform value-based care and efforts to reduce non-value added healthcare utilization [15].

Recent research suggests that cancer survivors spend considerable time interacting with the healthcare system. Across populations, these time burdens can vary considerably, with some studies in advanced gastrointestinal cancer suggesting that individuals interact with the healthcare system 1 in every 4 days, despite a median survival of under 6 months [9]. Similarly, other studies in advanced stage cancer [16–18] and among those on cancer clinical trials [19] demonstrate healthcare contact on more than 30% of days. Even within a broad population of individuals with a cancer history [20], survivors spent nearly one month over the course of a year receiving healthcare outside the home. While these studies highlight the growing evidence of the time burden of healthcare contact among survivors, many of these studies focus on individuals with advanced stage disease, specific cancer types, or evaluate time burdens within a single institution or year [9, 16, 17, 19, 20]. We have less information about the longer-term trajectory of the time burdens experienced across a broad population of cancer survivors and how these burdens compare to those without a cancer diagnosis. Understanding how time burdens vary across the trajectory of the cancer continuum for a broad range of cancer survivors can provide key insights into the unique timepoints and populations most impacted by these burdens, informing future interventions to drive value-based cancer care.

Our aims were to a) describe trajectories of healthcare contact after a cancer diagnosis relative to those without cancer, and b) describe key sociodemographic and cancer characteristics associated with higher levels of healthcare contact after a cancer diagnosis.

## Materials and Methods

### Study Population

This study includes individuals enrolled in the Cancer Prevention Study-II Nutrition Cohort (CPS-II NC), a prospective observational study from the American Cancer Society [21]. Participants received biennial questionnaires beginning in 1997 on demographics, lifestyle and cancer incidence (described in detail elsewhere [21]). Briefly, this cohort included adults aged 40 and over at their baseline survey who resided in 21 states with population-based cancer registries, allowing for verification of self-reported cancer diagnoses and death information through linkage to the cancer registries, the National Death Index, or medical record review [22]. CPSII-NC data were linked with Medicare data from 1999–2017. Among individuals in this linkage, we identified cancer cases diagnosed with invasive disease from 1/1999–6/2017. Included were individuals diagnosed after their Medicare coverage start date and were required to be enrolled in fee-for-service Medicare two years prior to diagnosis (to assess comorbidity) until death or the end of study follow-up. We then matched these individuals 1:4 with controls (no cancer history according to CPS-II NC) on age, sex and two-year continuous Medicare enrollment prior to diagnosis (for cases) or pseudo-diagnosis (for controls). From this population, we created two time-periods to examine healthcare contact at key points after cancer diagnosis: 1) the year of diagnosis (2 months prior to 12 months post-diagnosis) and 2) the last year of life (last 12 months prior to death). These two time periods were selected as individuals would likely experience the most intensive screening, diagnostic and treatment management during this time relative to other periods in the cancer continuum [14].

### Measures

Our primary outcome was healthcare contact days, derived using a previously published algorithm from Gupta et. al [20]., that creates a hierarchical count of distinct days with healthcare contact during each time-period using claims from the Medicare inpatient, outpatient, physician, and skilled nursing facility files. Briefly, we looked across all types of healthcare claims for individuals and classified them as having a healthcare contact day if they had a bill for healthcare services on a unique day. Care received and billed in multiple settings over the course of a day (e.g., inpatient hospitalization, clinic visit) was classified as a single healthcare contact day. Details on variables and applicable codes for identifying and classifying healthcare contact days by claim type can be found in prior work by our team [20].

From the CPS-II NC, we also assessed self-reported gender (woman, man), race/ethnicity (Non-Hispanic White, Other), age at diagnosis or pseudo-diagnosis (for controls), and rural–urban residence (based on rural–urban commuting area (RUCA) classification; metropolitan (RUCA 1–3), micropolitan (RUCA 4–6), rural/small town (RUCA 7–10)) [23] in the closest survey prior to their diagnosis. Cancer type (hematologic, lung, breast, prostate, colorectal, other) and stage (local, regional/distant) at diagnosis was verified from cancer registry, medical record and death certificate information [21]. We calculated the Charlson comorbidity Index based on diagnosis codes in the Medicare claims from the two years prior to diagnosis using previously published algorithms [24, 25]. Finally, we identified receipt of chemotherapy and radiation within 6 months of diagnosis in Medicare files using established methods and updated with Healthcare Common Procedure Coding System and International Classification of Disease codes [26–29].

### Analysis

For each time-period (year of diagnosis and last year of life), we used Chi-squared tests to examine differences in demographics between cases and controls. We then evaluated the median percentage of days with healthcare contact each month during both time-periods in cases versus controls. Finally, we used logistic regression to examine the relationship between sociodemographic and cancer characteristics and being in the highest quartile of percentage of healthcare contact days during each time-period, overall and by cancer stage (local, regional/distant). Two-sided *p*-values < 0.05 were considered statistically significant. All analyses were conducted using SAS V9.4. All aspects of CPS-II and its linkage with Medicare data were approved by the Emory Institutional Review Board.

## Results

We identified 13,510 cases and 54,040 controls during their initial year of diagnosis (for cases) or pseudo-diagnosis (for controls). The majority of the cohort was aged 70–80 years (57.7%), male (55.3%), non-Hispanic White (97.9%), with ≥ 1 comorbidity (51.2%), lived in a metropolitan area (78.5%) and survived over one-year post-diagnosis (median survival 24.5 months) (Table 1). Matched controls had slightly fewer comorbidities than cases (no comorbidities: 49.3% controls vs. 46.8% cases, Appendix 1a). Among cases, the most common cancer types were prostate (24.0%), breast (14.9%), hematologic (12.7%), and lung (10.5%); 39.4% were diagnosed with regional/distant stage disease and 52.9% received chemotherapy and/or radiation (Appendix 1a). In the last year of life cohort, demographic differences emerged, with cases during this period more likely to be female, younger and with fewer comorbidities relative to controls (Appendix 1b).

**Table 1:** Association between sociodemographic and cancer characteristics and being in the highest quartile of percentage of days with healthcare contact in the year of diagnosis and last year of life among individuals in the Cancer Prevention Study Nutrition Cohort (CPS-NC)

Variable		Initial Year of Diagnosis^+^										Last Year of Life^+^									
		Number of Individuals 67,550 (%)	Total Cohort			Local Stage Cases			Regional/Distant Stage Cases			Number of Individuals 16,738 (%)	Total Cohort			Local Stage Cases			Regional/Distant Stage Cases		
			OR	95% CI		OR	95% CI		OR	95% CI			OR	95% CI		OR	95% CI		OR	95% CI	
Cancer Type	Control	54,040 (80.0)	Ref			Ref			Ref			10,655 (63.7)	Ref			Ref			Ref		
	Other	3,905 (5.8)	5.80	5.32	6.32	3.84	3.43	4.30	13.20	11.12	15.67	2,134 (12.7)	2.07	1.84	2.34	1.50	1.22	1.85	2.58	2.19	3.03
	Colorectal	1,221 (1.8)	7.61	6.58	8.80	5.78	4.73	7.06	11.39	8.98	14.45	566 (3.4)	1.93	1.59	2.34	1.53	1.06	2.19	2.25	1.76	2.88
	Prostate	3,237 (4.8)	2.28	2.04	2.54	2.11	1.87	2.39	5.19	3.93	6.87	881 (5.3)	0.87	0.71	1.07	0.74	0.56	0.97	1.88	1.27	2.78
	Breast	2,018 (3)	2.86	2.52	3.25	2.77	2.40	3.20	4.21	3.13	5.66	419 (2.5)	0.75	0.57	1.01	0.60	0.40	0.89	1.18	0.76	1.83
	Lung	1,412 (2.1)	14.47	12.14	17.25	11.44	8.57	15.26	17.43	13.63	22.29	1,098 (6.6)	2.70	2.32	3.14	1.22	0.83	1.78	3.49	2.91	4.18
	Hematologic	1,717 (2.5)	7.54	6.59	8.63	5.66	4.02	7.97	7.95	6.81	9.27	985 (5.9)	3.26	2.80	3.81	2.15	1.29	3.60	3.56	3.00	4.22
Treatment	No Chemotherapy/Radiation*	60,393 (89.4)	Ref			Ref			Ref			13,443 (80.4)	Ref			Ref			Ref		
	Chemotherapy Only	2,329 (3.4)	9.96	8.61	11.52	4.95	3.93	6.24	11.48	9.25	14.23	1,412 (8.4)	1.25	1.08	1.44	1.19	0.79	1.79	1.10	0.93	1.31
	Radiation Only	3,545 (5.2)	3.87	3.47	4.32	3.78	3.32	4.30	5.40	4.09	7.12	1,106 (6.6)	1.29	1.09	1.53	1.34	1.03	1.75	1.41	1.10	1.81
	Chemotherapy + Radiation	1,283 (1.9)	31.10	23.99	40.32	21.25	15.21	29.69	40.59	25.39	64.88	777 (4.6)	1.40	1.17	1.66	1.24	0.83	1.83	1.29	1.04	1.60
Age	65- < 70	6,085 (9)		Ref			Ref			Ref		897 (5.4)		Ref			Ref			Ref	
	70- < 75	17,450 (25.8)	1.21	1.10	1.33	1.24	1.12	1.38	1.16	1.04	1.30	3,203 (19.1)	1.02	0.85	1.22	0.92	0.73	1.15	0.99	0.82	1.21
	75- < 80	21,545 (31.9)	1.57	1.43	1.72	1.62	1.46	1.78	1.54	1.38	1.71	5,270 (31.5)	1.06	0.89	1.26	1.01	0.82	1.25	1.03	0.85	1.24
	80- < 85	15,345 (22.7)	1.89	1.72	2.08	1.94	1.76	2.15	1.89	1.70	2.10	4,641 (27.7)	1.03	0.86	1.22	0.96	0.77	1.19	1.01	0.84	1.22
	85 +	7,125 (10.5)	2.07	1.87	2.29	2.11	1.89	2.36	2.03	1.81	2.28	2,727 (16.3)	0.81	0.68	0.98	0.72	0.57	0.90	0.78	0.63	0.95
Gender	Women	30,195 (44.7)	Ref			Ref			Ref			5,808 (34.7)	Ref			Ref			Ref		
	Men	37,355 (55.3)	0.94	0.89	0.98	0.94	0.89	0.98	0.95	0.91	1.00	10,930 (65.3)	0.94	0.87	1.02	0.93	0.84	1.03	0.97	0.89	1.05
Race/Ethnicity	Non-Hispanic White	66,112 (97.9)	Ref			Ref			Ref			16,455 (98.3)	Ref			Ref			Ref		
	Other	1,438 (2.1)	0.71	0.61	0.82	0.67	0.57	0.79	0.68	0.57	0.80	283 (1.7)	0.99	. 0.75	1.32	0.93	0.66	1.32	1.01	0.74	1.36
Comorbidities	None	32,956 (48.8)	Ref			Ref			Ref			5,090 (30.4)	Ref			Ref			Ref		
	1 Comorbidity	16,592 (24.6)	2.29	2.16	2.42	2.36	2.22	2.50	2.42	2.27	2.58	4,265 (25.5)	1.38	1.24	1.53	1.36	1.19	1.56	1.38	1.23	1.55
	2 Comorbidities	8,360 (12.4)	4.31	4.04	4.59	4.44	4.15	4.75	4.51	4.20	4.85	2,786 (16.6)	1.90	1.70	2.13	2.00	1.73	2.30	1.82	1.60	2.07
	3 + Comorbidities	9,642 (14.3)	10.57	9.95	11.23	10.92	10.26	11.63	11.36	10.64	12.13	4,597 (27.5)	2.94	2.66	3.25	3.34	2.96	3.78	3.04	2.73	3.39
Residence^	Metropolitan	53,001 (78.5)	Ref			Ref			Ref			13,135 (78.5)	Ref			Ref			Ref		
	Micropolitan	6,672 (9.9)	0.87	0.80	0.93	0.87	0.81	0.94	0.86	0.79	0.93	1,647 (9.8)	1.02	0.90	1.15	1.03	0.89	1.20	0.99	0.86	1.13
	Rural/Small Town	7,877 (11.7)	0.73	0.68	0.79	0.75	0.69	0.81	0.72	0.66	0.78	1,956 (11.7)	0.87	0.77	0.98	0.87	0.75	1.00	0.85	0.75	0.97
Median survial in months (Interquartile range)			24.5 (7.1–56.3)			29.6 (10.2–62.3)			22.5 (6.8–50.8)												

^+^Initial Year of Diagnosis: 2 months prior to 12 months post-diagnosis or pseudo-diagnosis; Last Year of Life: Last 12 Months of Life; *No chemotherapy/radiation includes both controls without cancer and cases with no chemotherapy/radiation reciept. In the year of diagnosis, 6,353 (9.4%) of the cohort had cancer but did not recieve chemotherapy/radiation, while in the last year of life 2,083 (12.4%) of cases recieved no chemotherapy/radiation. ^Residence (Metropolitan: Rural–Urban Commuting Area (RUCA): 1–3), (Micropolitan: RUCA 4–6), (Rural/Small Town: RUCA 7–10)

**Fig. 1:**
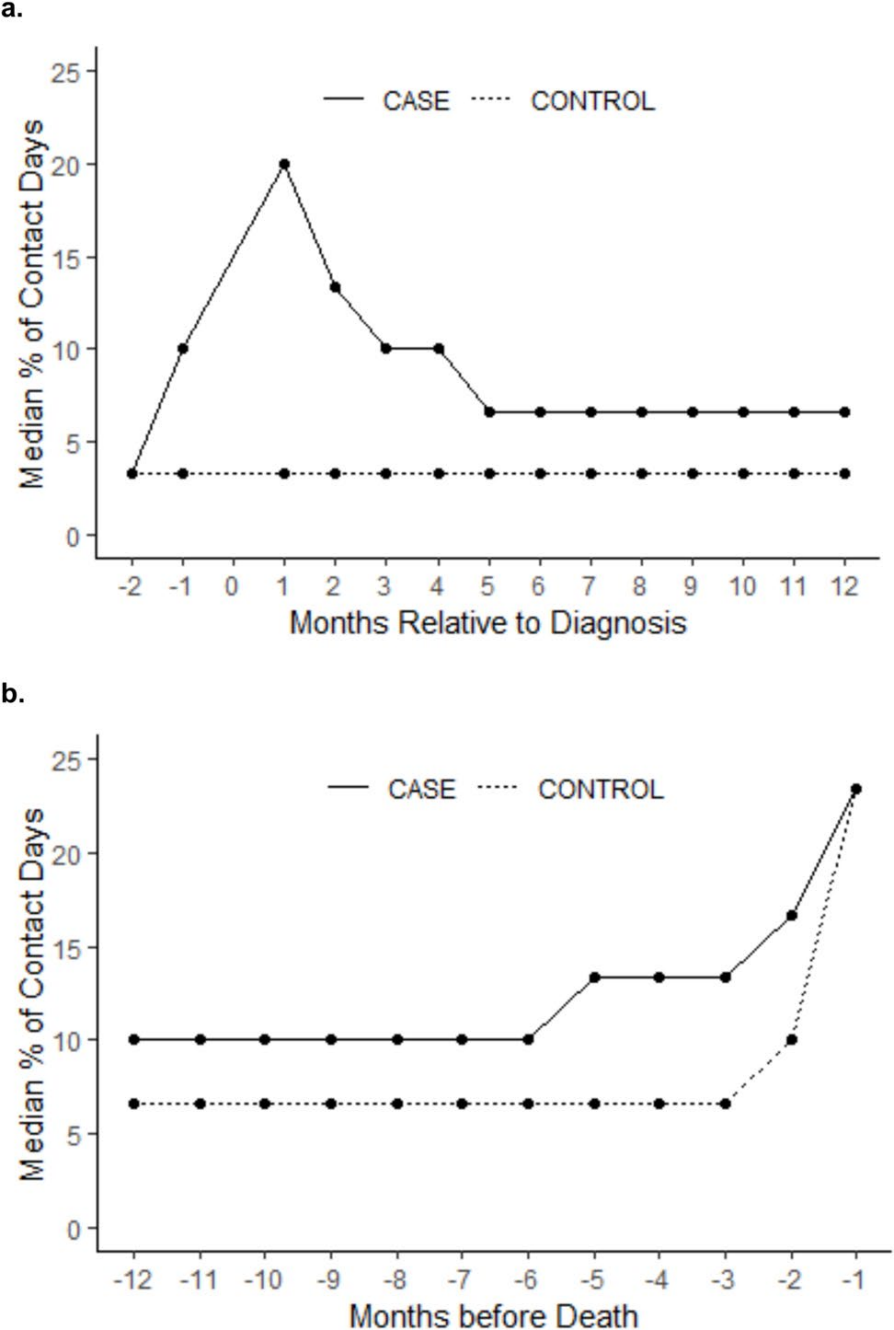
Legend: Median Percentage of Contact Days for Cancer Cases and Controls in the a) Year of Diagnosis^+^ and b) Last Year of Life, Cancer Prevention Study Nutrition Cohort (CPS-NC) II; Notes: Contact days: count of distinct days with healthcare contact [20] during each month. ^+^Year of Diagnosis: 2 months prior to 12 months post diagnosis or pseudo-diagnosis. **a** Month 0 is the month of diagnosis. **b** Month 0 is the month of death

**Fig. 2:**
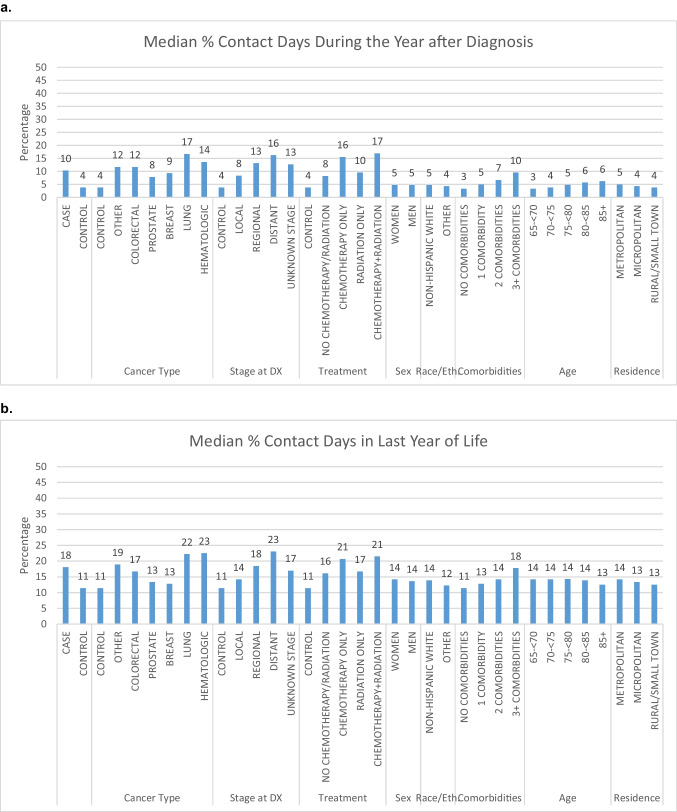
Legend. Median Percentage of Contact Days for Cancer Cases and Controls by Sociodemographic and Cancer Characteristics in the a) Year of Diagnosis^+^ and b) Last Year of Life, Cancer Prevention Study Nutrition Cohort (CPS-NC) Notes: Contact days: count of distinct days with healthcare contact [20] during the year of diagnosis (**a**) and last year of life (**b**). ^+^Year of Diagnosis: 2 months prior to 12 months post-diagnosis or pseudo-diagnosis; Last Year of Life: last 12 months prior to death; Eth.: Ethnicity

For all cancer cases, the median percentage of days with healthcare contact spiked at 20% in the month of diagnosis compared with 4% among controls (Fig. 1a), remaining higher than controls throughout the year after diagnosis. Cases diagnosed with lung or hematologic cancers (Median % contact days (lung: 17%, hematologic cancer: 14%, control 4%), those with regional/distant stage disease (distant: 16%, regional: 13%, control: 4%), those receiving chemotherapy and/or radiation (chemotherapy only: 16%, radiation only: 10%, chemotherapy + radiation: 17%, control: 4%) and those with > 3 comorbidities (> 3 comorbidities: 10%, no comorbidities: 3%) all had a higher median % of contact days vs. controls (Fig. 2a). Multivariable models showed similar patterns (Table 1), with lung and hematologic cancers (Odds Ratio (OR), 95% Confidence Interval (CI): 14.47 (12.14, 17.25) lung cancer case vs. control), those receiving chemotherapy and/or radiation (OR (95% CI): 31.10 (23.99, 40.32): chemotherapy + radiation vs. control), older individuals (OR (95% CI): 2.07 (1.87, 2.29): aged 85 + vs. age 65–70), females (OR (95% CI): 0.94 (0.89, 0.98): men vs. women), those with a higher number of comorbidities (OR (95% CI): 10.57 (9.95, 11.23): 3 + comorbidities vs. no comorbidities), Non-Hispanic White race/ethnicity (OR (95% CI): 0.71 (0.61, 0.82): other race/ethnicity vs. Non-Hispanic White), and living in a metropolitan area (OR (95% CI): 0.73 (0.68, 0.79), rural/small town vs. metropolitan) having the largest odds of being in the highest quartile of percentage of healthcare contact days (Table 1).

By the end of follow-up, 24.8% of the cohort (*N* = 16,738) had died (Table 1). In the last year of life, cancer cases had a higher median percentage of contact days vs. controls for all months except for the month of death, when contact days were similar (Fig. 1b). Cases had a larger increase in the median percentage of contact days in the months leading up to death relative to controls. Over the last year of life, cases had a median % of healthcare contact days of 18% vs. 11% among controls. This equates to approximately 1 in 5 days with healthcare contact in the last year of life among cancer cases (Fig. 2b). Unadjusted (Fig. 2b) and multivariable models (Table 1) both showed highest levels of healthcare contact in the last year of life among lung and hematologic cancers (OR (95% CI):3.26 (2.80, 3.81) hematologic cancer case vs. control), those receiving chemotherapy and/or radiation (OR (95% CI): 1.40 (1.17, 1.66): chemotherapy + radiation vs. control), older individuals (with the exception of the oldest age group who had lower odds of healthcare contact; OR (95% CI): 1.03 (0.86, 1.22): aged 80–85 vs. age 65–70), those with more comorbidities (OR, (95% CI): 2.94 (2.66, 3.25) 3 + comorbidities vs. no comorbidities) and living in metropolitan areas (OR (95% CI): 0.87 (0.77, 0.98), rural/small town vs. metropolitan). No significant differences were identified by gender or race/ethnicity. Sensitivity analyses stratifying by stage at diagnosis (Table 1) as well as limiting to those who survived 12 months (data not shown) during each time period showed similar associations.

## Discussion and Conclusion

We identified substantial healthcare contact in the year after diagnosis as well as in the last year of life among cancer survivors relative to controls. In the year after diagnosis, cancer cases experience a spike in healthcare contact, when individuals are likely to receive diagnostic work-up, develop and deploy cancer-directed treatments, and manage treatment-related conditions [5, 6]. However, healthcare contact remains substantially higher among cases vs. controls beyond the initial months after diagnosis, extending into the final year of life, even though many in CPS-II are longer-term survivors. Of note, approximately 1 in 5 days in the last year of life is spent with healthcare contact among cases (an excess of 25 days of healthcare contact relative to controls). However, we find high variability in healthcare contact after diagnosis and last year of life, with the highest contact burdens in those with lung and hematologic cancers, later stage disease and more extensive treatment modalities (e.g., chemotherapy and radiation).

Our work echoes the emerging research on treatment and logistic burdens after diagnosis [8, 9, 12, 20], extending prior work to examine our understanding of the trends in burden of healthcare contact across a broad range of cancer types, stages and treatment modalities relative to those without a cancer diagnosis. Previous work has highlighted the extensive time burdens those with advanced stage disease experience, despite expected survival of less than six months in many cases. Across this literature, those with stage IV gastrointestinal cancer [9, 17] and stage IV non-small cell lung cancer [16] experienced healthcare contact on more than 1 in 3 days after diagnosis. High levels of healthcare contact have been demonstrated in broader populations of cancer survivors as well. In a recent analysis of community dwelling older adults with a history of cancer, survivors spent almost one month in a single calendar year receiving healthcare outside the home [20]. Importantly, these patterns have been seen across clinical settings (e.g., academic health centers, Veteran’s Administration hospitals) in both the US and Canada. Consistent with prior patterns of healthcare contact, our study demonstrated the highest healthcare contact in the first six months after diagnosis and last 6 months of life, similar to the U-shaped curve of healthcare use among cancer survivors seen in prior studies [16, 17]. Our work builds on these patterns to demonstrate how these spikes in healthcare contact compare to those without a cancer diagnosis in broader populations of cancer survivors, providing evidence to inform treatment selection and planning discussions between healthcare providers and patients in the first year of diagnosis and beyond. While many aspects of care at these critical points in the cancer continuum are essential to improved survival (e.g., infusions, treatment planning and monitoring), emerging research has highlighted challenges and potential inefficiencies with oncology care delivery (e.g., scheduling oncology activities on multiple days vs. back-to-back appointments) that may increase the perceived burdens of these activities [12]. As a result, understanding populations most impacted by greater healthcare contact after diagnosis can inform development of new policies (e.g., multi-specialty clinics, coordinated scheduling) to address these burdens.

Not all populations experienced high levels of healthcare contact in our study. While individuals with local stage disease still experienced higher healthcare contact relative to those without a cancer history in the first year after diagnosis (Median % contact days: 8% local; 4% control), much of this added healthcare contact is likely to contribute to improved long-term survival and be perceived as value-added contact to improve outcomes [8]. Alternatively, healthcare contact was highest in individuals with distant stage disease (almost 1 in 5 days in the last year of life). This aligns with prior work demonstrating the highest time burdens at the end of life. One prior study reported that, even among advanced stage patients (who survived fewer than 3 months), almost 40% days in the month after diagnosis were spent receiving healthcare (e.g., hospitalized) [9]. We additionally found significantly higher healthcare contact by treatment modality and cancer type, with the highest burdens in those with multi-modal therapy and lung and hematologic cancers. This is not surprising as lung cancers are commonly diagnosed at later stages and hematologic cancers often include multi-modal therapies or therapies that extend over longer time periods [14, 30]. These burdens may also be greater for individuals managing comorbid conditions that may require alternate or modified treatment modalities [31]. We also found lower healthcare contact in rural populations, potentially resulting from differential choices of therapies requiring fewer healthcare contact days (e.g., mastectomy vs. breast conserving surgery with radiation), but also potentially lower receipt of guideline concordant care, as has been shown in prior studies [32, 33]. This may exacerbate disparities in these communities, particularly for socioeconomically disadvantaged populations [34], and should be evaluated further in future work. By providing data on broader patterns of healthcare contact across disease stages, types and patient demographics, we can inform discussions and decision making that is most consistent with a cancer survivor’s goals of care while advancing health equity across populations.

Going forward, our work can inform future development of new programs and policies. First, our study can inform discussions between healthcare providers, patients and care partners about the expected time commitments most applicable to their cancer type, stage and treatment modality. However, we recognize this as a starting point for conversations, considering the emerging literature highlighting that significant healthcare demands placed on patients exist outside healthcare settings (e.g., traveling to and waiting for appointments, home care tasks, scheduling, paperwork) that may result in considerable additional time burdens for patients and their families [15]. Future work should further characterize these burdens and identify populations most impacted by the administrative, logistic, financial and time burdens [8] to develop targeted interventions to promote equity in access and outcomes after diagnosis. Second, as new therapies are developed to drive improved survival and outcomes across cancer populations, consideration should be taken for the added time burdens for not only participating on these therapeutic trials [19], but also the tradeoffs between additional healthcare contact and survival under these new regimens. Emerging work is examining the potential for incorporating time burden as a clinical trial end point and should be explored in future work [35]. Finally, developing targeted interventions for individuals most impacted by time burdens in our study is warranted. Prior work has shown that frequent, uncoordinated clinic appointments (e.g., care appointments on multiple days across clinic sites) are especially burdensome for individuals, particularly when considering travel, parking and waiting time [36]. Additionally, administrative and logistic burdens are particularly challenging given complexities with prior approvals and other paperwork (e.g., paid leave). Developing value-based, patient-centered care models that take into consideration preferences for home-based care when feasible, tailored scheduling, care coordination and logistic support may begin to address these challenges.

We recognize certain limitations in the current work. First, we focus on two key time periods of healthcare contact after diagnosis rather than the entire cancer continuum. We see reductions in healthcare contact at the end of the first year of diagnosis and a ramp up to higher contact in the final months of life, suggesting a U-shaped curve similar to prior studies [16, 17]. Additionally, we do not know the reasons underlying specific healthcare contact and whether these days are value-added from the perspective of the healthcare provider or patient. Future work should continue to explore when and how healthcare contact best meets the goals of care with minimizing patient and care partner time burdens.

This work can identify populations with significant healthcare burden and may present opportunities to develop interventions that improve quality of life and outcomes for individuals with cancer and their families. These findings can serve as a framework for discussion between providers and patients and inform interventions to reduce non-value added healthcare contact at the end-of-life as well as opportunities to reduce time burden during the year after diagnosis.

## Supplementary Information

Below is the link to the electronic supplementary material.

ESM 1 (DOCX 54.4 KB)

## Data Availability

Data are available from the American Cancer Society by following the ACS Data Access Procedures (https://www.cancer.org/research/population-science/research-collaboration.html) for researchers who meet the criteria for access to confidential data. Please email cohort.data@cancer.org to inquire about access.

## References

[1] Siegel RL, Kratzer TB, Wagle NS, Sung H, Jemal A (2026) Cancer statistics, 2026. CA Cancer J Clin 76(1):e70043. 10.3322/caac.70043 41528114 10.3322/caac.70043 PMC12798275

[2] Tonorezos E, Devasia T, Mariotto AB et al (2024) Prevalence of cancer survivors in the United States. J Natl Cancer Inst 116(11):1784–1790. 10.1093/jnci/djae135 39002121 10.1093/jnci/djae135 PMC11542986

[3] Shapiro CL (2018) Cancer survivorship. N Engl J Med 379(25):2438–2450. 10.1056/NEJMra1712502 30575480 10.1056/NEJMra1712502

[4] Kent EE, Park EM, Wood WA, Bryant AL, Mollica MA (2021) Survivorship Care of Older Adults With Cancer: Priority Areas for Clinical Practice, Training, Research, and Policy. J Clin Oncol 39(19):2175–2184. 10.1200/jco.21.00226 34043450 10.1200/JCO.21.00226 PMC8260922

[5] Gupta A, Eisenhauer EA, Booth CM (2022) The Time Toxicity of Cancer Treatment. J Clin Oncol 40(15):1611–1615. 10.1200/jco.21.02810 35235366 10.1200/JCO.21.02810

[6] Gupta A, Jensen EH, Virnig BA, Beg MS (Apr2022) Time-Related Burdens of Cancer Care. JCO Oncol Pract 18(4):245–246. 10.1200/op.21.00662 34709950 10.1200/OP.21.00662

[7] Hall ET, Sridhar D, Singhal S et al (May2021) Perceptions of time spent pursuing cancer care among patients, caregivers, and oncology professionals. Support Care Cancer 29(5):2493–2500. 10.1007/s00520-020-05763-9 32935204 10.1007/s00520-020-05763-9

[8] Parsons HM, Gupta A, Jewett P, Vogel RI (2025) The intersecting time, administrative, and financial burdens of a cancer diagnosis. J Natl Cancer Inst 117(4):595–600. 10.1093/jnci/djae252 39392423 10.1093/jnci/djae252 PMC11972685

[9] Patel VR, Ramesh V, Tsai AK et al (Nov2023) Health Care Contact Days Experienced by Decedents With Advanced GI Cancer. JCO Oncol Pract 19(11):1031–1038. 10.1200/op.23.00232 37738532 10.1200/OP.23.00232 PMC10667015

[10] Fu SJ, Rose L, Dawes AJ, Knowlton LM, Ruddy KJ, Morris AM (2021) Out-of-Pocket Costs Among Patients With a New Cancer Diagnosis Enrolled in High-Deductible Health Plans vs Traditional Insurance. JAMA Netw Open 4(12):e2134282–e2134282. 10.1001/jamanetworkopen.2021.34282 34935922 10.1001/jamanetworkopen.2021.34282 PMC8696568

[11] Shih YT, Xu Y, Liu L, Smieliauskas F (2017) Rising Prices of Targeted Oral Anticancer Medications and Associated Financial Burden on Medicare Beneficiaries. J Clin Oncol 35(22):2482–2489. 10.1200/jco.2017.72.3742 28471711 10.1200/JCO.2017.72.3742 PMC5536165

[12] Gupta A, Johnson WV, Henderson NL et al (2024) Patient, caregiver, and clinician perspectives on the time burdens of cancer care. JAMA Netw Open 7(11):e2447649. 10.1001/jamanetworkopen.2024.47649 39602118 10.1001/jamanetworkopen.2024.47649 PMC12040224

[13] Sav A, King MA, Whitty JA et al (Jun2015) Burden of treatment for chronic illness: a concept analysis and review of the literature. Health Expect 18(3):312–324. 10.1111/hex.12046 23363080 10.1111/hex.12046 PMC5060781

[14] National Comprehensive Cancer Network. NCCN Guidelines. https://www.nccn.org/guidelines/category_1. Accessed 3 May 2026

[15] Vogel RI, Jewett P, Parsons H et al (2025) Time burden in patients with metastatic breast and ovarian cancer from clinic and home demands. JAMA Netw Open 8(12):e2549957. 10.1001/jamanetworkopen.2025.49957 41400954 10.1001/jamanetworkopen.2025.49957 PMC12709373

[16] Gupta A, Nguyen P, Kain D et al (2024) Trajectories of health care contact days for patients with stage IV non-small cell lung cancer. JAMA Netw Open 7(4):e244278. 10.1001/jamanetworkopen.2024.4278 38587847 10.1001/jamanetworkopen.2024.4278 PMC11002696

[17] Johnson WV, Phung QH, Patel VR et al (2024) Trajectory of Healthcare Contact Days for Veterans With Advanced Gastrointestinal Malignancy. Oncologist 29(2):e290–e293. 10.1093/oncolo/oyad313 38016182 10.1093/oncolo/oyad313 PMC10836304

[18] Johnson WV, Hsu ML, Gupta A (Sep2025) A Fifth of Their Days: The Time Commitments of Advanced Cancer and Its Care. JCO Oncol Pract 21(9):1232–1234. 10.1200/op-24-01085 39818966 10.1200/OP-24-01085

[19] Gupta A, Hay AE, Crump M et al (2023) Contact days associated with cancer treatments in the CCTG LY.12 trial. Oncologist 28(9):799–803. 10.1093/oncolo/oyad128 37226534 10.1093/oncolo/oyad128 PMC10485297

[20] Gupta A, Chant ED, Mohile S et al (2024) Health care contact days among older cancer survivors. JCO Oncol Pract 20(7):943–952. 10.1200/op.23.00590 38452315 10.1200/OP.23.00590 PMC11268556

[21] American Cancer Society. Cancer Prevention Study II (CPS-II). https://www.cancer.org/research/population-science/cancer-prevention-and-survivorship-research-team/acs-cancer-prevention-studies/cancer-prevention-study-2.html. Accessed 11 Oct 2024

[22] National Cancer Institute. Cancer Prevention Study II Nutrition Cohort (CPS-II Nutrition). https://cedcd.nci.nih.gov/cohort?id=110. Accessed 5 May 2026

[23] US Department of Agriculture. Rural-urban commuting area codes- descriptions and maps. https://www.ers.usda.gov/data-products/rural-urban-commuting-area-codes/descriptions-and-maps. Accessed 1 May 2026

[24] National Cancer Institute. SEER-Medicare: Comorbidity SAS Macros. https://healthcaredelivery.cancer.gov/seermedicare/considerations/calculation.html. Accessed 15 Oct 2024

[25] National Cancer Institute. Comorbidity SAS macro (2021 version). https://healthcaredelivery.cancer.gov/seermedicare/considerations/macro-2021.html. Accessed 1 May 2026

[26] National Cancer Institute. Observational Research in Oncology Toolbox: CanMed. https://seer.cancer.gov/oncologytoolbox/canmed/hcpcs/?q=&hcpcs=&seerrxcategory=Chemotherapy. Accessed 5 May 2026

[27] National Cancer Institute. CanMED: Cancer Medications Enquiry Database. https://seer.cancer.gov/oncologytoolbox/. Accessed 17 Dec 2024

[28] Virnig BA, Warren JL, Cooper GS, Klabunde CN, Schussler N, Freeman J (2002) Studying radiation therapy using SEER-Medicare-linked data. Med Care 40:Iv-49–54. 10.1097/00005650-200208001-00007 10.1097/00005650-200208001-00007 12187168

[29] Warren JL, Harlan LC, Fahey A, et al. Utility of the SEER-Medicare data to identify chemotherapy use. *Med Care*. Aug 2002;40(8 Suppl):Iv-55–61. 10.1097/01.mlr.0000020944.17670.d7 10.1097/01.MLR.0000020944.17670.D7 12187169

[30] American Cancer Society. Cancer Facts & Figures 2026. https://www.cancer.org/content/dam/cancer-org/research/cancer-facts-and-statistics/annual-cancer-facts-and-figures/2026/2026-cancer-facts-and-figures.pdf. Accessed 13 April 2026

[31] George M, Smith A, Sabesan S, Ranmuthugala G (2021) Physical comorbidities and their relationship with cancer treatment and its outcomes in older adult populations: systematic review. JMIR Cancer 7(4):e26425. 10.2196/26425 34643534 10.2196/26425 PMC8552093

[32] Wercholuk AN, Parikh AA, Snyder RA (Sep2022) The Road Less Traveled: Transportation Barriers to Cancer Care Delivery in the Rural Patient Population. JCO Oncol Pract 18(9):652–662. 10.1200/op.22.00122 35834768 10.1200/OP.22.00122

[33] Longacre CF, Neprash HT, Shippee ND, Tuttle TM, Virnig BA (2021) Travel, Treatment Choice, and Survival Among Breast Cancer Patients: A Population-Based Analysis. Womens Health Rep (New Rochelle) 2(1):1–10. 10.1089/whr.2020.0094 33786524 10.1089/whr.2020.0094 PMC7957915

[34] Bhatia S, Landier W, Paskett ED et al (2022) Rural-Urban Disparities in Cancer Outcomes: Opportunities for Future Research. J Natl Cancer Inst 114(7):940–952. 10.1093/jnci/djac030 35148389 10.1093/jnci/djac030 PMC9275775

[35] Gupta A, Nguyen P, Wilson BE, Booth CM, Hanna TP (2025) Health care contact days and outcomes in clinical trials vs routine care among patients with non-small cell lung cancer. JAMA Netw Open 8(4):e255033. 10.1001/jamanetworkopen.2025.5033 40232720 10.1001/jamanetworkopen.2025.5033 PMC12000967

[36] Johnson WV, Valisekka SS, Ogunleye OO et al (2025) Patient-, care partner-, and clinician-proposed solutions to address the time toxicity of cancer care. Support Care Cancer 33(11):965. 10.1007/s00520-025-09954-0 41107599 10.1007/s00520-025-09954-0 PMC12534262

