# Supplementary material for: Healthcare contact days in cancer survivors relative to those with no cancer history
Source: Support Care Cancer. 2026 Jun 16;34(7):664. doi: 10.1007/s00520-026-10884-8 (PMC13272605; doi:10.1007/s00520-026-10884-8)
Supplement: Supplementary file 1 — (DOCX 54.4 KB) [file 520_2026_10884_MOESM1_ESM.docx]

**Appendix 1. Demographics and Cancer Characteristics (when applicable) of the Included Cohort, Cancer Prevention Study Nutrition Cohort (CPS-NC) II**

**1a. Year of Diagnosis^+^ Included Cohort (N=67,550)**

| VARIABLE |  | TOTAL COHORT | CONTROLS | CANCER CASES | P-VALUE |
| --- | --- | --- | --- | --- | --- |
|  |  | N=67,550 | N=54,040 | N=13,510 |  |
|  |  |  |  |  |  |
|  |  | N (%) | N (%) | N (%) |  |
|  |  |  |  |  |  |
| GENDER |  |  |  |  |  |
|  | WOMEN | 30,195 (44.7) | 24,156 (44.7) | 6,039 (44.7) | p = 1.00 |
|  | MEN | 37,355 (55.3) | 29,884 (55.3) | 7,471 (55.3) |  |
|  |  |  |  |  |  |
| RACE/ETHNICITY |  |  |  |  |  |
|  | NON-HISPANIC WHITE | 66,112 (97.9) | 52,876 (97.8) | 13,236 (98) | p = 0.36 |
|  | OTHER | 1,438 (2.1) | 1,164 (2.2) | 274 (2) |  |
|  |  |  |  |  |  |
| COMORBITITY |  |  |  |  |  |
|  | NONE | 32,952 (48.8) | 26,628 (49.3) | 6,324 (46.8) | p < 0.0001 |
|  | 1 COMORBIDITY | 16,589 (24.6) | 13,130 (24.3) | 3,459 (25.6) |  |
|  | 2 COMORBIDITIES | 8,364 (12.4) | 6,638 (12.3) | 1,726 (12.8) |  |
|  | 3+ COMORBDITIES | 9,645 (14.3) | 7,644 (14.1) | 2,001 (14.8) |  |
|  |  |  |  |  |  |
| AGE |  |  |  |  |  |
|  | 65-<70 | 6,085 (9) | 4,868 (9) | 1,217 (9) | p = 1.00 |
|  | 70-<75 | 17,450 (25.8) | 13,960 (25.8) | 3,490 (25.8) |  |
|  | 75-<80 | 21,545 (31.9) | 17,236 (31.9) | 4,309 (31.9) |  |
|  | 80-<85 | 15,345 (22.7) | 12,276 (22.7) | 3,069 (22.7) |  |
|  | 85+ | 7,125 (10.5) | 5,700 (10.5) | 1,425 (10.5) |  |
|  |  |  |  |  |  |
| CANCER TYPE |  |  |  |  |  |
|  | CONTROL | 54,040 (80) | 54,040 (100) | 0 (0) | - |
|  | OTHER | 3,905 (5.8) | 0 (0) | 3,905 (28.9) |  |
|  | COLORECTAL | 1,221 (1.8) | 0 (0) | 1,221 (9) |  |
|  | PROSTATE | 3,237 (4.8) | 0 (0) | 3,237 (24) |  |
|  | BREAST | 2,018 (3) | 0 (0) | 2,018 (14.9) |  |
|  | LUNG | 1,412 (2.1) | 0 (0) | 1,412 (10.5) |  |
|  | HEMATOLOGIC | 1,717 (2.5) | 0 (0) | 1,717 (12.7) |  |
|  |  |  |  |  |  |
| CANCER STAGE |  |  |  |  |  |
|  | CONTROL | 54,040 (80) | 54,040 (100) | 0 (0) | - |
|  | LOCAL | 7,369 (10.9) | 0 (0) | 7,369 (54.5) |  |
|  | REGIONAL | 2,367 (3.5) | 0 (0) | 2,367 (17.5) |  |
|  | DISTANT | 2,960 (4.4) | 0 (0) | 2,960 (21.9) |  |
|  | UNKNOWN | 814 (1.2) | 0 (0) | 814 (6) |  |
|  |  |  |  |  |  |
| TREATMENT |  |  |  |  |  |
|  | CONTROL | 54,040 (80) | 54,040 (100) | 0 (0) | p < 0.0001 |
|  | NO RADIATION/  CHEMOTHERAPY | 6,353 (9.4) | 0 (0) | 6,353 (47) |  |
|  | CHEMOTHERAPY ONLY | 2,329 (3.4) | 0 (0) | 2,329 (17.2) |  |
|  | RADIATION ONLY | 3,545 (5.2) | 0 (0) | 3,545 (26.2) |  |
|  | CHEMOTHERAPY+  RADIATION | 1,283 (1.9) | 0 (0) | 1,283 (9.5) |  |
|  |  |  |  |  |  |
| RESIDENCE^*^ |  |  |  |  |  |
|  | METROPOLITAN (1-3) | 53,000 (78.5) | 42,244 (78.2) | 10,756 (79.6) | p = 0.0005 |
|  | MICROPOLITAN (4-6) | 6,674 (9.9) | 5,379 (10) | 1,295 (9.6) |  |
|  | RURAL/SMALL TOWN (7-10) | 7,876 (11.7) | 6,417 (11.9) | 1,459 (10.8) |  |

Notes: Contact days: count of distinct days with healthcare contact^9^ during the year of diagnosis. +Initial Year of Diagnosis: 2 months prior to 12 months post-diagnosis or pseudo-diagnosis; *Residence (Metropolitan: Rural-Urban Commuting Area (RUCA): 1-3), (Micropolitan: RUCA 4-6), (Rural/Small Town: RUCA 7-10).

**1b. Last Year of Life^#^ Included Cohort (N=16,738)**

| VARIABLE |  | TOTAL COHORT | CONTROLS | CANCER CASES | P-VALUE |
| --- | --- | --- | --- | --- | --- |
|  |  | N=16,738 | N=10,655 | N=6,083 |  |
|  |  |  |  |  |  |
|  |  | N (%) | N (%) | N (%) |  |
|  |  |  |  |  |  |
| GENDER |  |  |  |  |  |
|  | WOMEN | 5,808 (34.7) | 3,284 (30.8) | 2,524 (41.5) | p < 0.0001 |
|  | MEN | 10,930 (65.3) | 7,371 (69.2) | 3,559 (58.5) |  |
|  |  |  |  |  |  |
| RACE/ETHNICITY |  |  |  |  |  |
|  | NON-HISPANIC WHITE | 16,455 (98.3) | 10,472 (98.3) | 5,983 (98.4) | p = 0.72 |
|  | OTHER | 283 (1.7) | 183 (1.7) | 100 (1.6) |  |
|  |  |  |  |  |  |
| COMORBITITY |  |  |  |  |  |
|  | NONE | 5,086 (30.4) | 2,874 (27) | 2,212 (36.4) | p < 0.0001 |
|  | 1 COMORBIDITY | 4,264 (25.5) | 2,658 (24.9) | 1,606 (26.4) |  |
|  | 2 COMORBIDITIES | 2,794 (16.7) | 1,828 (17.2) | 966 (15.9) |  |
|  | 3+ COMORBDITIES | 4,594 (27.4) | 3,295 (30.9) | 1,299 (21.4) |  |
|  |  |  |  |  |  |
| AGE |  |  |  |  |  |
|  | 65-<70 | 897 (5.4) | 494 (4.6) | 403 (6.6) | p < 0.0001 |
|  | 70-<75 | 3,200 (19.1) | 1,901 (17.8) | 1,299 (21.4) |  |
|  | 75-<80 | 5,275 (31.5) | 3,376 (31.7) | 1,899 (31.2) |  |
|  | 80-<85 | 4,639 (27.7) | 3,022 (28.4) | 1,617 (26.6) |  |
|  | 85+ | 2,727 (16.3) | 1,862 (17.5) | 865 (14.2) |  |
|  |  |  |  |  |  |
| CANCER TYPE |  |  |  |  |  |
|  | CONTROL | 10,655 (63.7) | 10,655 (100) | 0 (0) | p < 0.0001 |
|  | OTHER | 2,134 (12.7) | 0 (0) | 2,134 (35.1) |  |
|  | COLORECTAL | 566 (3.4) | 0 (0) | 566 (9.3) |  |
|  | PROSTATE | 881 (5.3) | 0 (0) | 881 (14.5) |  |
|  | BREAST | 419 (2.5) | 0 (0) | 419 (6.9) |  |
|  | LUNG | 1,098 (6.6) | 0 (0) | 1,098 (18.1) |  |
|  | HEMATOLOGIC | 985 (5.9) | 0 (0) | 985 (16.2) |  |
|  |  |  |  |  |  |
| CANCER STAGE |  |  |  |  |  |
|  | CONTROL | 10,655 (63.7) | 10,655 (100) | 0 (0) | p < 0.0001 |
|  | LOCAL | 2,083 (12.4) | 0 (0) | 2,083 (34.2) |  |
|  | REGIONAL | 1,191 (7.1) | 0 (0) | 1,191 (19.6) |  |
|  | DISTANT | 2,224 (13.3) | 0 (0) | 2,224 (36.6) |  |
|  | UNKNOWN | 585 (3.5) | 0 (0) | 585 (9.6) |  |
|  |  |  |  |  |  |
| TREATMENT |  |  |  |  |  |
|  | CONTROL | 10,655 (63.7) | 10,655 (100) | 0 (0) | p < 0.0001 |
|  | NO RADIATION/  CHEMOTHERAPY | 2,788 (16.7) | 0 (0) | 2,788 (45.8) |  |
|  | CHEMOTHERAPY ONLY | 1,412 (8.4) | 0 (0) | 1,412 (23.2) |  |
|  | RADIATION ONLY | 1,106 (6.6) | 0 (0) | 1,106 (18.2) |  |
|  | CHEMOTHERAPY+  RADIATION | 777 (4.6) | 0 (0) | 777 (12.8) |  |
|  |  |  |  |  |  |
| RESIDENCE^*^ |  |  |  |  |  |
|  | METROPOLITAN (1-3) | 13,134 (78.5) | 8,308 (78) | 4,826 (79.3) | p = 0.11 |
|  | MICROPOLITAN (4-6) | 1,648 (9.8) | 1,069 (10) | 579 (9.5) |  |
|  | RURAL/SMALL TOWN (7-10) | 1,956 (11.7) | 1,278 (12) | 678 (11.1) |  |

Notes: Contact days: count of distinct days with healthcare contact^9^ during the last year of life Last Year of Life: Last 12 Months of Life; *Residence (Metropolitan: Rural-Urban Commuting Area (RUCA): 1-3), (Micropolitan: RUCA 4-6), (Rural/Small Town: RUCA 7-10).
